# Impact of perineural invasion on the outcome of patients with synchronous colorectal liver metastases treated with neoadjuvant chemotherapy and surgery

**DOI:** 10.1007/s12094-023-03138-0

**Published:** 2023-04-07

**Authors:** Gabriel Zozaya, Javier Álvarez Cienfuegos, Pablo Martí-Cruchaga, José Luis Hernández-Lizoain, Jorge Baixauli, Fernando Pardo, Ramón Robledano, Javier Rodríguez, Leire Arbea, Fernando Rotellar

**Affiliations:** 1 Department of General Surgery, School of Medicine, Clínica Universidad de Navarra, University of Navarra, Av. Pío XII, 36, 31008 Pamplona, Spain; 2 Institute of Health Research of Navarra (IdisNA), Pamplona, Spain; 3 CIBER Fisiopatología de la Obesidad y Nutrición (CIBERobn), Instituto de Salud Carlos III, 31008 Pamplona, Spain; 4 Department of Pathology, School of Medicine, Clínica Universidad de Navarra, University of Navarra, Pamplona, Spain; 5 Department of Medical Oncology, School of Medicine, Clínica Universidad de Navarra, University of Navarra, Pamplona, Spain; 6 Department of Radiation Oncology, School of Medicine, Clínica Universidad de Navarra, University of Navarra, Pamplona, Spain

**Keywords:** Colorectal cancer, Liver metastases, Neoadjuvant chemotherapy, Perineural invasion, Stage IV

## Abstract

**Purpose:**

To analyze the prognostic value of variables of the primary tumor in patients with synchronous liver metastases in colorectal cancer (CLRMs) treated with neoadjuvant chemotherapy and surgery.

**Methods/Patients:**

From a prospective database, we retrospectively identified all patients with synchronous CLRMs who were treated with neoadjuvant chemotherapy and liver resection. Using univariate and multivariate analyses, we identified the variables associated with tumor recurrence. Overall survival and disease-free survival were calculated using the Kaplan–Meier method with differences determined by the Cox multiple hazards model. Results were compared using the log-rank test.

**Results:**

Ninety-eight patients with synchronous CLRMs were identified. With a median follow-up of 39.8 months, overall survival and disease-free survival at 5 and 10 years were 53%, 41.7%, 29% and 29%, respectively. Univariate analysis identified three variables associated with tumor recurrence: location in the colon (*p* = 0.025), lymphovascular invasion (*p* = 0.011) and perineural invasion (*p* = 0.005). Multivariate analysis identified two variables associated with worse overall survival: perineural invasion (HR 2.36, 95% CI 1.162–4.818, *p* = 0.018) and performing frontline colectomy (HR 3.286, 95% CI 1.256–8.597, *p* = 0.015). Perineural invasion remained as the only variable associated with lower disease-free survival (HR 1.867, 95% CI 1.013–3.441, *p* = 0.045). Overall survival at 5 and 10 years in patients with and without perineural invasion was 68.2%, 54.4% and 29.9% and 21.3%, respectively (HR 5.920, 95% CI 2.241–15.630, *p* < 0.001).

**Conclusions:**

Perineural invasion in the primary tumor is the variable with most impact on survival in patients with synchronous CLRMs treated with neoadjuvant chemotherapy and surgery.

## Introduction

Colorectal cancer is the second leading cause of cancer deaths in men and women and it is estimated that 14.5–25% of patients present metastases at the time of diagnosis (synchronous) [1, 2]. Of these, 15–40% are resectable [2, 3].

Over the last two decades, there has been an extraordinary improvement in oncologic outcomes from the surgical treatment of colorectal liver metastases (CLRM)—both synchronous (CRLMs) and metachronous—with a 5-year overall survival (OS) and disease-free survival (DFS) of 50–60% and 38–40%, respectively[3–5].

Given that patients with CLRMs constitute a heterogeneous group and with the aim of selecting patients with a better prognosis and personalizing treatment, multiple prognostic scores have been developed [6–8]. In these scores, most variables relate to the degree of liver involvement—the number of metastases, their uni- or bi-lobular location and size—and to the liver surgery but do not take into account significant histologic parameters of the primary tumor [6, 9, 10].

For this reason, several authors have questioned the clinical usefulness of these scores and have put greater emphasis on the importance of the histologic characteristics of the primary tumor as well as new chemotherapy regimens [9–11].

In previous articles, we and other authors have reported the greater prognostic and predictive value of perineural invasion (PNI) in the clinical outcomes of cancer of the colon and rectum [9, 12–15]. A 50% reduction in survival has been reported in patients with PNI [13].

The aim of the present study is to analyze the predictive value of the histologic characteristics of the primary tumor (PNI and lymphovascular invasion) in patients with synchronous liver metastases treated with neoadjuvant chemotherapy (NCT) and liver resection.

## Materials and methods

### Study design, population and endpoints

From a prospective institutional database of all patients treated for primary colorectal adenocarcinoma, we retrospectively identified those with synchronous liver metastases (stage IV) treated with systemic NCT and liver resection. In the cases of locally advanced cancer of the rectum, we included external radiotherapy using a technique described elsewhere [15].

Patients under the age of 18 were excluded as were patients with metastases with other histologic origins, tumors that were considered irresectable, those that did not receive NCT or those who had some type of non-liver disease.

The study was approved by the center’s Research Ethics Committee (protocol number 2022.043) and was carried out following the norms of the latest version of the Helsinki Declaration.

Surgical indication and the sequencing of the surgery of the primary tumor and metastases were decided consensually in a multi-disciplinary session including medical oncologists, hepatobiliary and colorectal surgeons, radiotherapists, radiologists and pathologists. Pre-operative staging was carried out using contrast-enhanced computed tomography (CT) of the thorax, abdomen and pelvis and magnetic resonance imaging (MRI) of the liver. In patients with doubtful lesions, positron emission tomography (PET) was performed following the guidelines of the National Comprehensive Cancer Network (NCCN) [16].

The patients underwent different NCT chemotherapy regimens based on FOLFOX (leucovorin + 5-Fluorouracil + oxaliplatin) or FOLFIRI (leucovorin + 5-Fluorouracil + CPT-11) or a “triplet” triple regimen based on oxaliplatin, irinotecan and capecitabine, or XELOX (oxaliplatin + capecitabine) with a cetuximab or bevacizumab regimen.

In patients with locally advanced rectal cancer, chemoradiotherapy was administered (50.4 Gy external-beam radiotherapy and infusional 5-Fluorouracil) [17].

Most liver resections were performed laparoscopically following the criteria of Lousville and Southampton and according to the Brisbane nomenclature. In all operations, intra-operative ultrasound was used to verify the number of metastases and their relationship with vessels and the remnant parenchyma.

Patients were followed up in the Department of Surgery following the recommendations of the National Comprehensive Cancer Network (NCCN) [17, 18] with a CT scan of the chest, abdomen and pelvis every 3–6 months in the first 2 years, and then every 6–12 months for up a total of 5–10 years. At each clinical review, carcinoembryonic antigen (CEA) levels were measured.

Any lesion with characteristics of liver relapse or metastatic disease was considered recurrence [16].

### Histopathologic analysis of the primary tumor

Specimens from the colon and rectum were studied using the protocol of the American College of Pathologists (CAP) and were staged using the criteria of the American Joint Committee on Cancer (AJCC), TNM classification (8th edition) [19]. The degree of differentiations was calculated using the three categories of the World Health Organization (WHO) classification (5th edition).

Perineural invasion was defined as the invasion of tumor cells in, around and through the nerve sheath (epineurium, perineurium, and endoneurium) or tumor in the perineural space that involved at least one third of the nerve circumference [20]: Lymphovascular invasion (LVI) was defined as the presence of tumor cells, identification of endothelial cells lining the space and attachment of tumor cells to the vascular wall.

The histologic response to the neoadjuvant treatment was evaluated using the criteria of the College of American Pathologists (CAP), in three categories: 0–1 (complete response or near complete response, score-1), 2 (partial response, score-2) and grade 3 (poor response or no response, score-3) [19].

The resection margin was defined as the distance from the lesion to the nearest resection margin. In patients with several metastases, the reference point taken was the lesion nearest the resection margin.

Overall survival (OS) was defined as the time from surgery to the date of last follow-up or death for any cause. Disease-free survival (DFS) was defined as the time from surgery to recurrence or death for any reason.

### Endpoints

The primary end-point of the study was disease-free survival (DFS), while the secondary end-point was overall survival (OS).

### Statistical analysis

Categorical variables are presented as frequencies and proportions (%). Continuous variables are reported using means (Standard Deviation, SD) or medians and ranges.

Categorical variables were analyzed using Pearson’s Chi-squared tests. Univariate analysis was performed to assess for any significant difference in clinicopathological parameters that influenced disease recurrence and survival following liver resection. Independent variables with a *p* value < 0.05 in the univariate analysis were entered into a logistic Cox regression (stepwise forward model). The Kaplan–Meier method was used to assess actuarial and disease-free survival. The survival curves obtained for the different categories of a factor were compared with the log-rank test.

Statistical analysis was performed using the SPSS Statistical software package for Windows version 16.0 (SPSS, Inc, Chicago, IL, USA) *p* values < 0.05 were considered to be statistically significant.

## Results

**Fig. 1:**
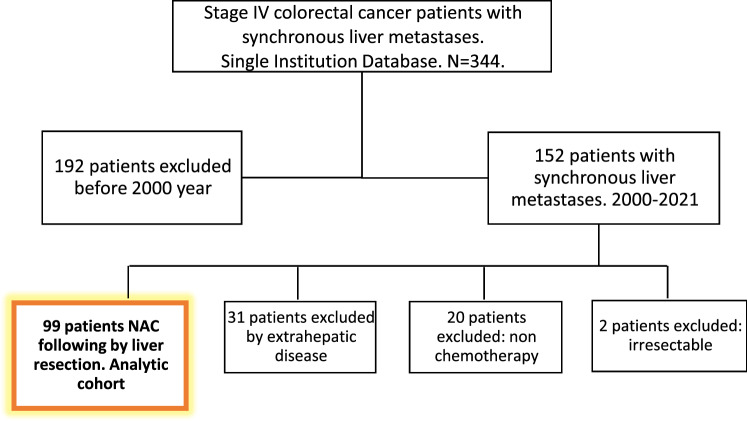
Flow diagram of the study. A total of 344 patients were registered. Finally, 99 patients were included in the analysis. NAC Neoadjuvant chemotherapy

Over the study period from a total of 344 patients with synchronous liver metastases 98 patients with CLRM treated with systemic NCT and liver resection were identified (Fig. 1). Table 1 summarizes the clinical and pathologic characteristics of the series.

**Table 1:** Demographic and clinical characteristics of the study patients

Variable	No patients	%
Sex		
Male	64	65.3
Female	34	34.7
Age (*y)*, mean (SD)	60.1	10.7
ASA score		
II	33	35.5
III	51	54.8
IV	9	9.7
Location of tumor		
Rectum	43	43.9
Left colon	38	38.8
Right colon	17	17.3
Pathological T classification		
T1	2	2.0
T2	14	14.3
T3	64	65.3
T4	12	12.2
Pathological N classification		
N0	36	36.7
N1	38	38.8
N2	24	24.5
Tumor differentiation		
Well	5	5.1
Moderate	59	60.2
Poor	7	7.1
Missing	27	27.6
Lymphovascular invasion	39	40.2
Perineural invasion	32	33.3
No of metastases, mean (SD)	3.8	2.5
No of liver metastases		
< 3	61	62.2
> 3	37	37.8
NACT regimen		
Triplet	49	52.1
Oxaliplatin-based	21	22.3
Irinotecan-based	5	5.3
Xeloda	19	20.2
Width of resection margin (cm), mean (SD)	4.3	6.3
No of lymph nodes retrieved, mean (SD)	15.0	7.8
Longest tumor size (cm), mean (SD)	3.1	1.8
Primary tumor regression score (CAP)		
0–1 near complete	42	42.9
2 partial response	40	40.8
3 poor response	16	16.3
Sequencing of treatment		
Simultaneous	25	25.5
Primary-first approach	45	45.9
Liver-first approach	28	28.6

*SD* standard deviation, *ASA* American Society of Anesthesiologists, *NACT* neoadjuvant chemotherapy, *CAP* American College of Pathologists

Sixty-four (65.3%) were male and the mean age was 60.1 years (SD, 10.7). In 43 patients (43.9%), the primary tumor was located in the rectum and in 55 (56.1%), in the colon—38 (38.8%) in the left colon and 17 (17.3%) in the right colon.

The mean number of liver metastases was 3.8 (SD, 2.5) but in 37 cases, the number of metastases was greater than 3. In 25 patients (25.5%), the hepatectomy and the resection of the tumor were performed simultaneously, in 45 (45.9%), resection of the primary tumor was performed first and in 28 (28.6%), the hepatectomy was performed first.

Analyses of KRAS and BRAF mutations and microsatellite instability were not performed in all cases as this was not part of routine clinical practice until a few years ago. In those cases in which such analyses were conducted, 16 of 29 patients presented KRAS mutations, 1 of 19 BRAS mutations and 3 of 34 microsatellite instability.

All patients received NCT—triplet in 49 patients (52.1%), in 21 (22.3%) a regimen based on Oxaliplatin, 5 (5.3%) with Irinotecan and 19 (20.2%) with Xeloda.

Laparoscopic hepatectomy was performed in 59 patients (60.2%) and mean hospital stay was 6.6 days (range 3–36).

Most of the primary tumors (*n* = 59, 60.2%) were moderately differentiated; although due to the neoadjuvant treatment, this parameter was not recorded in 27 patients (27.6%). Forty-two patients (42.9%) showed a good response to treatment (6 with a complete response and 26 with a moderate response). In 40 patients (40.8%), a minimum response was observed and in 16 (16.3%), no response to treatment was observed.

Thirty-two patients (33.3%) presented perineural invasion and 39 (40.2%) lymphovascular invasion. PNI in the colon and rectum was observed in 19 (34.5%) and 13 (31.7%) cases, respectively (*p* = 0.472) LVI in the colon and rectum was observed in 20 (36.4%) and 19 (45%) cases, respectively (*p* = 0.250).

### Outcome

**Fig. 2:**
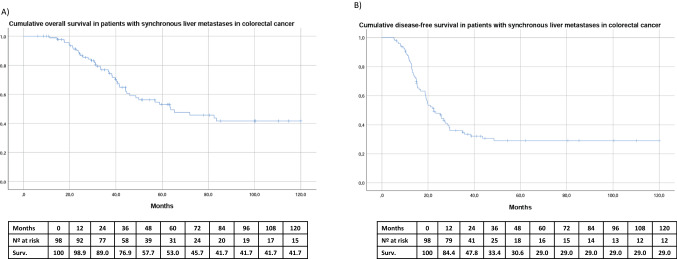
A Cumulative overall survival (OS) of patients with synchronous colorectal liver metastases treated with neoadjuvant chemotherapy and hepatectomy. 2B Cumulative disease-free survival (DFS) of patients with synchronous colorectal liver metastases treated with neoadjuvant chemotherapy and hepatectomy

With a median follow-up of 39.8 months, the median OS overall survival was 63.7 months (95% CI 33.4–93.9). The OS overall survival of the series at 5 and 10 years was 53% and 41.7%, respectively (Fig. 2A).

Sixty-four patients (65.3%) experienced tumor recurrence in the course of the follow-up, mostly in the liver and lung. Median DFS disease-free survival was 22.4 months and DFS disease-free survival at 5 and 10 years was 29% and 29%, respectively (Fig. 2B).

In the univariate analysis, 3 pathologic variables of the primary tumor were identified as being associated with relapse following hepatectomy: location in the colon (*p* = 0.025), lymphovascular invasion (*p* = 0.011) and perineural invasion (*p* = 0.005) (Table 2).

**Table 2:** Clinicopathological characteristics of patients with resected CLRMs by recurrence

	No recurrence	Recurrence	*P* value
	*N* = 34	*N* = 64	
Sex			
Male	21 (61.8)	43 (67.2)	
Female	13 (41.9)	20 (32.3)	0.375
Age	62.2	59.0	0.168
ASA score			
II	13 (41.9)	20 (32.3)	0.399
III	12 (45.2)	37 (59.7)	
IV	4 (12.9)	5 (8.1)	
BMI	26.3 (3.0)	25.9 (4.2)	0.580
Location of tumor			
Rectum	20 (58.8)	23 (35.9)	0.025
Colon	14 (41.2)	41 (64.1)	
Lymphovascular invasion	8 (23.5)	31 (49.2)	0.011
Perineural invasion	5 (15.2)	27 (42.9)	0.005
Tumor differentiation			
Well	3 (8.8)	2 (3.1)	0.625
Moderate	19 (55.9)	40 (62.5)	
Poor	2 (5.9)	5 (7.8)	
Missing	10 (29.4)	17 (26.6)	
Tumor depth			
T0	4 (11.8)	2 (3.1)	0.081
T1	2 (5.9)	0 (0.0)	
T2	5 (14.7)	9 (14.1)	
T3	21 (61.8)	43 (67.2)	
T4	2 (5.9)	10 (15.6)	
N classification			
N0	13 (38.2)	23 (35.9)	0.494
N1	15 (44.1)	23 (35.9)	
N2	6 (17.6)	18 (28.1)	
Primary tumor regression score			
0–1 near complete	17 (50.0)	25 (39.1)	0.086
2 partial response	9 (26.5)	31 (48.4)	
3 poor response	8 (23.5)	8 (12.5)	
Number of metastases			
< 3	24 (70.6)	37 (57.8)	0.153
> 3	10 (29.4)	27 (42.2)	
Size of metastases, *mm*	25.1 (25.1)	25.0 (21.61)	0.975
Surgical sequence			
Simultaneous	13 (38.2)	12 (48.0)	0.097
Primary-first approach	12 (35.3)	33 (51.6)	
Liver-first approach	9 (26.5)	19 (29.7)	

*CLRMs* colorectal cancer liver metastases, *ASA* American Society of Anesthesiologists, *BMI* body mass index (calculated as weight in kilograms divided by height in meters squared)

In the multivariate Cox regression analysis, two variables were identified as being negatively associated with OS overall survival: PNI (HR 2.36, 95% CI 1.162–4.818, *p* = 0.018) and performing colectomy first (HR 3.286, 95% CI 1.256–8.597, *p* = 0.015 (Table 3). In contrast, for DFS disease-free survival, only perineural invasion remained as a negative prognostic factor (HR 1.867, 95% CI 1.013–3.441, *p* = 0.045) (Table 4) (Fig. 2).

**Table 3:** Multivariate analysis of prognostic factors associated with overall survival following hepatectomy for synchronous CLRMs

Variable	HR	95%CI	*P* value
Location of tumor			
Rectum	1		
Colon	1.405	0.823–2.398	0.213
Perineural invasion	1.867	1.013–3.441	0.045
Lymphovascular invasion	1.336	0.765–2.334	0.309
Surgical sequence			
Simultaneous	1		
Primary-first approach	1.967	0.936–4.131	0.074
Liver-first approach	1.620	0.765–2.334	0.309

*CRLMs* colorectal cancer liver metastases, *HR* hazard ratio, *CI* confidence interval

**Table 4:** Multivariate analysis of prognostic factors associated with disease-free survival following hepatectomy for synchronous CLRMs

Variable	HR	95%CI	*P* value
Location of tumor			
Rectum	1		
Colon	0.833	0.436–1.590	0.579
Perineural invasion	2.366	1.162–4.818	0.018
Lymphovascular invasion	1.049	0.542–2.028	0.888
Surgical sequence			
Simultaneous	1		
Primary-first approach	3.286	1.256–8.597	0.015
Liver-first approach	1.951	0.636–5.990	0.123

*CRLMs* colorectal cancer liver metastases, *HR* hazard ratio, *CI* confidence interval

**Fig. 3:**
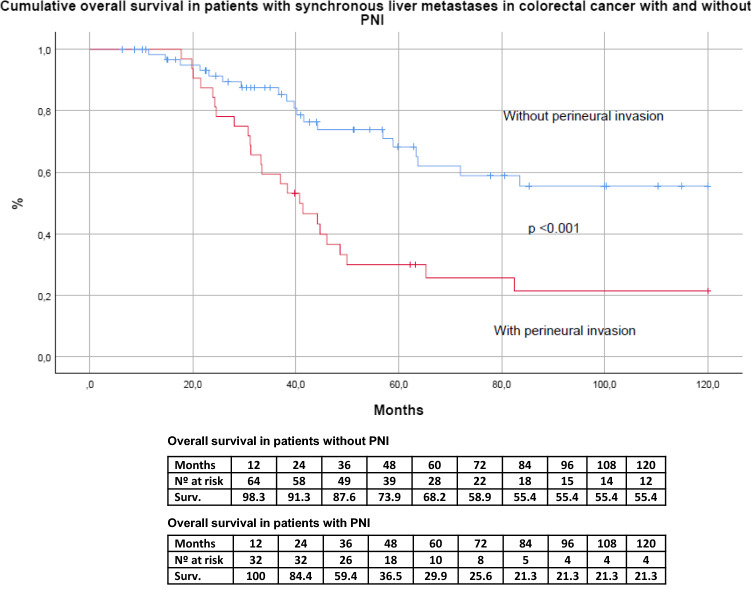
Cumulative overall survival (OS) curve estimated by the method of Kaplan–Meier of patients with synchronous colorectal liver metastases and hepatectomy comparing cases without (PNI−) and with perineural invasion (PNI +)

**Fig. 4:**
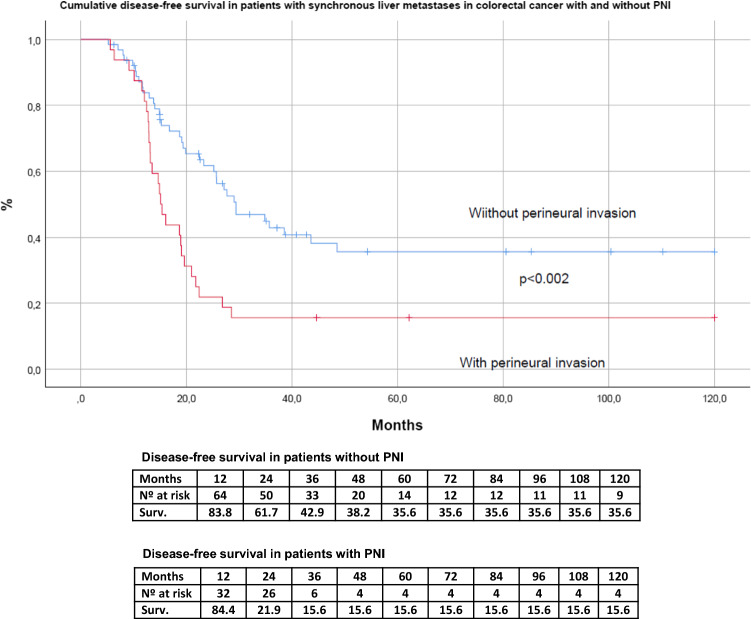
Cumulative disease-free survival (DFS) curve estimated by the method of Kaplan–Meier of patients with synchronous colorectal liver metastases and hepatectomy comparing cases without (PNI−) and with perineural invasion (PNI +)

Figures 3 and 4 show the impact of perineural invasion on OS overall survival and DFS in the series. The OS at 5 and 10 years in patients with no PNI was 68.2% and 55.4% respectively, while the OS for patients with PNI was 29.9% and 21.3% (HR 5.920. 95% CI 2.241–15.639, *p* =  < 0.001). The DFS at 5 and 10 years in patients with PNI was 15.6%, while the patients without PNI was 35,6% (HR 2.202; 95% CI 1.327–3.653, *p* =  < 0.002),

## Discussion

Approximately 25–30% of patients with colorectal cancer present with liver metastases at the time of diagnosis, of which only 15–40% are potentially resectable [5]

In these patients, the most frequently recommended treatment is neoadjuvant systemic chemotherapy and liver resection, either administered simultaneously or before or after resection of the primary tumor although controversy exists as to the sequential ordering of the surgery [3, 5].

Over the last two decades, notable advances have been made in oncologic outcomes following liver resection of synchronous metastases with an OS overall and DFS disease-free survival of 50% and 38–40%, respectively [3, 5].

In this period, many prognostic scores have been developed with the aim of identifying those patients with a better prognosis and obtaining better results [6–8].

However, some authors have highlighted that the majority of these scores are based on parameters related to the liver surgery—the number and size of the metastases, uni- or bi-lobular involvement, use of blood products, response to the NCT—but ignore important histologic aspects of the primary tumor [9, 10].

For this reason, we decided to analyze the relevant histologic variables of the primary tumor in the oncologic outcomes of a risk group undergoing liver resection of synchronous metastases in colorectal cancer.

In our series, the incidence of NPI was similar to that reported by Liebl et al. [21] and higher than that reported in cohorts of patients with less advanced tumors (13–18%), thus confirming the association of PNI with other markers of poor prognosis such as lymph node metastases, lymphovascular invasion and degree of differentiation [22].

Similarly, LVI (40.2%) was higher than in other series (10–14%) which included less advanced cases, highlighting the association between LVI and other histologic variables, tumor size, lymph node metastases and degree of differentiation and even a relationship with DFS [23].

Although reporting of both of these parameters—PNI and LVI—is recommended in the most recent clinical guidelines on colorectal cancer, great variability exists between studies depending on the histologic techniques used and the degree of specialization of the pathologist [24].

The OS and DFS obtained in our study are similar to those reported by other authors [6, 8, 9, 11] When studying the variables associated with tumor recurrence, we were surprised to find that the univariate analysis identified three variables associated with a worse prognosis: location of the primary tumor, lymphovascular invasion and perineural invasion.

However, in the multivariate analysis for OS, performing the colectomy first and PNI were shown to be independent risk factors. In contrast, in the multivariate analysis for DFS, only PNI remained as the sole risk factor for relapse. To the best of our knowledge, this is the first time that the negative impact of PNI in the primary tumor in the resection of liver metastases has been reported.

Liebig et al. [12] reported PNI as being a negative prognostic factor for colorectal cancer with a greater impact than either TNM or degree of differentiation. Subsequently, Albo et al. [13] and other authors, and among them our own group [14], confirmed the negative effect of PNI on survival in cancers of the colon and rectum. It has been calculated that the presence of PNI reduces survival in colorectal cancer by 50% [13, 24].

In this context, it is striking that Gomez et al. [10] in their analysis of the multiple prognostic factors related to survival after resection of liver metastases in 259 patients, found that PNI in the metastases was the most important negative prognostic factor (risk ratio 3.152, 96% CI 1.636–6.074, *p* < 0.001). So much so was this the case, that 5 years after the hepatectomy none of the patients with perineural invasion were still alive.

These findings confirm the previously reported results in which PNI was associated with other prognostic factors: TNM, degree of differentiation and lymphovascular invasion [12, 13, 21].

Although beyond the scope of the present study, recently excellent reviews have been published on neurogenesis and axogenesis and their relationship with the invasive character of several gastrointestinal tumors (of the stomach, pancreas and colon and rectum) [25].

Even at early stages, it has been reported that the release of neurotrophic factors including nerve growth factor, glial cell line-derived neurotrophic factor and brain-derived neurotrophic factor is an essential component of tumor progression [25].

Some authors have argued that as the rectum has greater autonomic enervation than the right colon, this could explain its greater tendency to produce metastases [13]. In our study, we found no differences in the degree of perineural invasion between the colon (34.5%) and the rectum (31.7%) or in lymphovascular invasion although this may well be because we were dealing with tumors at very advanced stages (IV).

### Limitations

In spite of the findings reported, we must mention some limitations to our study. First, although the data were collected prospectively, ours is a retrospective study and this may have introduced some bias in the selection of the patients.

Second, given the strict selection criteria—the resection of synchronous metastases—the cohort is relatively small which may explain why some parameters did not reach statistical significance.

Third, as the study was based on a single center with homogeneous treatment—neoadjuvant chemotherapy, surgical technique and intense follow-up—extrapolation of findings to other centers may be limited.

Finally, we are aware that the absence of molecular genetic variables such as RAS/BRAF-V600E mutations or data on circulating tumor DNA could explain the worse prognosis of some patients.

## Conclusions

Neoadjuvant chemotherapy and liver resection of synchronous liver metastases offer and a 5-year overall and disease-free survival of 53% and 29%, respectively.

Perineural invasion of the primary tumor is the most important prognostic factor for OS and DFS in synchronous liver metastases treated with neoadjuvant chemotherapy and surgical resection. Therefore, in patients with PNI in the primary tumor, it is essential to look for more specific chemotherapy regimens or other alternatives that bear this prognostic factor in mind.

## Data Availability

The data could be obtained from the corresponding author upon reasonable request.
